# Prediction of Stem Cell Mobilization Failure in Patients with Hodgkin and Non-Hodgkin Lymphoma

**DOI:** 10.4274/tjh.galenos.2020.2020.0409

**Published:** 2021-08-25

**Authors:** Haluk Demiroğlu, Rafiye Çiftçiler, Yahya Büyükaşık, Hakan Göker

**Affiliations:** 1 Hacettepe University Faculty of Medicine, Departments of Hematology, Ankara, Turkey

**Keywords:** Hodgkin lymphoma, Non-Hodgkin lymphoma, Stem cell mobilization, Mobilization failure

## Abstract

**Objective:**

Autologous stem cell transplantation (ASCT) is a significant and potentially curative treatment modality for patients with relapsed/refractory lymphoma. Insufficient mobilization and harvest of peripheral stem cells can be a major obstacle for performing ASCT. The aim of this study was to evaluate the factors that might influence mobilization failure in patients with lymphoma.

**Materials and Methods:**

Eighty-seven patients diagnosed with non-Hodgkin and Hodgkin lymphoma who underwent stem cell mobilization afterwards at the Hacettepe University Medical School Bone Marrow Transplantation Center, Turkey, between the years of 2000 and 2018 were evaluated.

**Results:**

A total of 87 patients were included in this study. In 66 of 87 patients (75.9%), the first mobilization trial was successful. Adequate (≥2x106/kg) CD34+ cells were collected in the first apheresis for 66 patients (9.5±8.1). For 21 of 87 (24.1%), the first mobilization trial was unsuccessful. Therefore, a second mobilization trial was performed for these patients with plerixafor (5.5±3.3). The number of CD34+ cells was significantly higher in patients who were successful in the first mobilization (p=0.002).

**Conclusion:**

The success rate of the first mobilization trial was found to be higher in patients with high platelet counts before mobilization and patients who received chemotherapy-based mobilization protocols. In the patients who had mobilization failure in the first trial, plerixafor was used in a later mobilization, and those patients had an adequate amount of stem cells for ASCT. Parameters predicting mobilization failure would allow for preemptive, more cost-effective use of such agents during the first mobilization attempt; however, risk factors for mobilization failure are still not clear.

## Abstract (tr)

**Amaç:**

Otolog kök hücre transplantasyonu (OKHT), relaps/refrakter lenfoma hastaları için önemli ve potansiyel olarak küratif bir tedavi yöntemidir. Yetersiz mobilizasyon ve periferik kök hücrelerin toplanması OKHT’nin gerçekleştirilmesi için büyük bir engel olabilir. Bu çalışmanın amacı lenfoma hastalarında mobilizasyon başarısızlığını etkileyebilecek faktörleri değerlendirmektir.

**Gereç ve Yöntemler:**

Hacettepe Üniversitesi Tıp Fakültesi Kemik İliği Nakil Merkezi’nde 2000-2018 yılları arasında Hodgkin ve non-Hodgkin lenfoma tanısı alan ve sonrasında kök hücre mobilizasyonu yapılan 87 hasta değerlendirildi.

**Bulgular:**

Bu çalışmaya toplam 87 hasta dahil edildi. Seksen yedi hastanın 66’sında (%75,9) ilk mobilizasyon denemesi başarılı oldu. 66 hastada (9,5±8,1) ilk aferezde yeterli (≥2x106/kg) CD34+ hücre toplandı. Seksen yedi kişiden 21’i (%24,1) için ilk mobilizasyon denemesi başarısız oldu. Bu nedenle bu hastalara pleriksafor ile (5,5±3,3) ikinci bir mobilizasyon denemesi yapıldı. İlk mobilizasyonda başarılı olan hastalarda CD34+ hücre sayısı anlamlı olarak yüksekti (p=0,002).

**Sonuç:**

Mobilizasyon öncesi trombosit sayısı yüksek olan hastalarda ve kemoterapi bazlı mobilizasyon protokolleri alan hastalarda ilk mobilizasyon denemesinin başarı oranı daha yüksek bulundu. İlk denemede mobilizasyon başarısızlığı olan hastalarda daha sonraki mobilizasyonda pleriksafor kullanılmış ve bu hastalarda OKHT için yeterli miktarda kök hücre mevcuttu. Mobilizasyon başarısızlığını öngören parametreler, ilk mobilizasyon girişimi sırasında bu tür ajanların önleyici, daha uygun maliyetli kullanımına izin verecektir; ancak, mobilizasyon başarısızlığı için risk faktörleri hala net değildir.

## Introduction

Autologous stem cell transplantation (ASCT) is a significant and potentially curative treatment modality for patients with relapsed/refractory lymphoma. However, 5%-40% of lymphoma patients fail to mobilize sufficient peripheral blood stem cells and thus cannot undergo ASCT, which is known to improve survival [1]. Hematopoietic stem cells generally circulate in very small numbers in the peripheral blood and have to be mobilized into the circulation prior to being collected by apheresis. Peripheral blood stem cell (PBSC) mobilization is accomplished by administration of granulocyte colony-stimulating factor (G-CSF) alone or in combination with chemotherapy [2]. Peripheral blood has been shown to be superior to bone marrow as a source of hematopoietic stem cells for ASCT [3]. Insufficient mobilization and harvest of peripheral stem cells can be a major obstacle for performing ASCT. Currently, a minimum of 2x10^6^ CD34+ cells/kg hematopoietic stem cells is considered appropriate in most centers to proceed to ASCT. This threshold is necessary for a rapid and sustained blood count recovery and for reduced hospitalization, blood product usage, and infections [4]. However, the optimal hematopoietic stem cell dose is about 5x10^6^/kg [5]. Bone marrow infiltration, advanced age, number of prior cytotoxic therapies, and myelodysplastic changes are the best defined factors associated with increased risk of mobilization failure [6,7].

We have collected and analyzed data from a series of non-Hodgkin and Hodgkin lymphoma patients who received ASCT in order to determine the frequency of harvest failure and to identify factors influencing PBSC mobilization outcomes. The aim of this study was to evaluate the factors that might influence mobilization failure in patients with lymphoma.

## Materials and Methods

### Study Design and Data Collection

This study was performed in a retrospective manner. Demographic data of the patients, treatment regimens, and stem cell mobilization data updates were obtained from the hospital database. As a result of the application standards of the hospitals of the Hacettepe University Medical School Bone Marrow Transplantation Center, Turkey, it has been recognized from the patient records that all of the studied patients had given informed consent at the time of hospitalization and before the administration of chemotherapy and other relevant diagnostic/therapeutic standards of care. Patients gave informed consent for procedures in accordance with the Declaration of Helsinki.

### Patients and Disease Characteristics

Eighty-seven patients diagnosed with non-Hodgkin and Hodgkin lymphoma who underwent stem cell mobilization afterwards at the Hacettepe University Medical School Bone Marrow Transplantation Center between the years of 2000 and 2018 were evaluated. The key inclusion criteria were patients ≥18 years of age diagnosed with non-Hodgkin or Hodgkin lymphoma who required systemic chemotherapy and underwent ASCT with Eastern Cooperative Oncology Group (ECOG) performance status (PS) of <2 [8] with an indication for ASCT.

Median age, gender, ECOG PS, lymphoma subtypes, stage at diagnosis, bone marrow infiltration at diagnosis, induction chemotherapy, salvage chemotherapy, chemotherapy cycles received before mobilization, radiotherapy before mobilization, platelet count before mobilization, mobilization protocols, and disease status before ASCT were compared for patients who had successful stem cell mobilization and those with stem cell mobilization failure. Additionally, disease status after ASCT, relapse rate, and mortality results were evaluated between these groups. The target CD34+ cell dose for collection was >2x10^6^/kg for each planned autograft. All patients received G-CSF at a dose of 10 µg/kg from day +5 until the peripheral stem cell harvest. CD34+ cells were measured in peripheral blood and apheresis products by flow cytometry. We had a CD34+ cut-off level of 20 µL for starting apheresis. We harvested the cells on the 5^th^ and/or 6^th^ day after beginning G-CSF administration. Peripheral blood CD34% and CD34/µL values at the first day on which leukocytes reached the value of 1x10^9^/L and were maintained above that threshold over at least 2 days were correlated with overall CD34+ collection. A harvest of less than 2x10^6^ CD34+/kg was considered as mobilization failure. Twenty-one patients received plerixafor as an additional mobilizing agent for the second apheresis. Subcutaneous plerixafor (0.24 mg/kg) was administered to the patients on the evenings of the 4^th^ and 5^th^ days of the mobilization protocol.

### Statistical Analysis

Statistical analyses were performed using SPSS 25 (IBM Corp., Armonk, NY, USA). Variables were investigated using visual (histograms, probability plots) and analytical (Kolmogorov-Smirnov/Shapiro-Wilk test) methods to determine whether they were normally distributed or not. Statistical comparisons were made using chi-square tests for categorical data. Student’s t-test for two independent samples was used for comparison of continuous numerical data. Variables found to be significant (p<0.05) in univariate analysis were tested in multivariate analysis, which was performed using a stepwise logistic regression model. Survival analyses were performed using the Kaplan-Meier test with log rank. Values of p<0.05 were considered statistically significant.

## Results

### Patient Characteristics

A total of 87 patients were included in this study. The median age was 48 (range: 18-70) years at the time of diagnosis. The baseline clinical and demographic characteristics of the patients are listed in Table 1. For 66 of 87 patients (75.9%), the first mobilization trial was successful. Adequate (≥2x10^6^/kg) CD34+ cells were collected in the first apheresis for 66 patients (9.5±8.1). For 21 of 87 (24.1%), the first mobilization trial was unsuccessful. Therefore, a second mobilization trial was conducted for these patients with plerixafor (5.5±3.3). The number of CD34+ cells was significantly higher in patients who were successful in the first mobilization (p=0.002). There were no differences in hematocrit at the time point of apheresis.

Between the two groups, there was no statistically significant gender (p=0.25) or age (p=0.07) difference. There was no significant difference between the ECOG PS of the patients (p=0.72). No significant difference was found between the groups in terms of lymphoma types (p=0.45). Number of chemotherapy cycles before stem cell mobilization was not statistically significantly different between patients who had mobilization failure and patients who had successful stem cell mobilization (p=0.78). The stages of both groups were similar at the time of diagnosis (p=0.69). There was no significant difference between bone marrow infiltration at diagnosis (p=0.24). There was no significant difference between the groups in terms of induction chemotherapy protocols (p=0.51). Platelet count before mobilization was higher in patients who had successful stem cell mobilization than in patients who had stem cell mobilization failure (p=0.041). After relapse, no significant difference was found between rescue chemotherapies given before mobilization (p=0.49). Disease status before ASCT was complete response (CR) in 27 (40.9%) patients, partial response (PR) in 28 (42.4%) patients, stable disease in 5 (7.6%) patients, and progressive disease in 6 (9.1%) patients in the successful mobilization group. Disease status before ASCT was CR in 8 (38.1%) patients, PR in 10 (47.6%) patients, stable disease in 1 (4.8%) patient, and progressive disease in 2 (9.5%) patients in the stem cell mobilization failure group for the first trial (p=0.95). The use of filgrastim or lenograstim as G-CSF did not affect mobilization success. There was no significant difference between the two groups in terms of filgrastim or lenograstim mobilization (p=0.20). However, when the patients who received only G-CSF or a chemotherapy-based mobilization protocol were evaluated, 19 (29.7%) of the patients who were mobilized with only G-CSF had mobilization failure, while only 2 (8.7%) patients who received a chemotherapy-based mobilization protocol had mobilization failure (p=0.04). This shows the superiority of chemotherapy-based mobilization.

### Post-transplant Outcomes

All of the patients finally underwent ASCT. Remarkably, disease status after ASCT (on day +100) was CR in 38 (61.3%) patients, PR in 1 (1.6%) patients, stable disease in 20 (32.3%) patients, and progressive disease in 3 (4.8%) patients in the successful mobilization group. Disease status after ASCT (on day +100) was CR in 13 (65%) patients, PR in 5 (5%) patients, stable disease in 4 (20%) patients, and progressive disease in 2 (10%) patients in the stem cell mobilization failure group for the first trial, as shown in Table 2. The relapse rate was significantly higher in patients who had stem cell mobilization failure than in those with successful stem cell mobilization (47.6% vs. 21.2%, p=0.01). Moreover, the mortality rate was significantly higher among patients who had stem cell mobilization failure than those with successful stem cell mobilization (38.1% vs. 16.7%, p=0.01).

### Overall Survival

**Figure 1:**
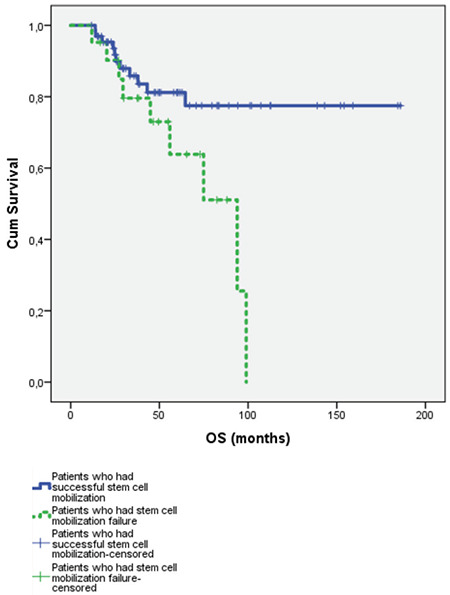
Overall survival (OS) of patients who had successful stem cell mobilization and patients who had stem cell mobilization failure (p=0.02).

The overall survival (OS) rate for patients who had successful stem cell mobilization was 151.6±9.3 months versus 71.4±7.8 months for patients with stem cell mobilization failure for the first trial; this was a statistically signiﬁcant difference, as shown in Figure 1 (p=0.02). The 3-year OS rates for patients with successful stem cell mobilization and those with stem cell mobilization failure for the first trial were 85% and 79%, respectively. The 5-year OS rates for patients with successful stem cell mobilization and stem cell mobilization failure for the first trial were 81% and 63%, respectively. OS was better in patients with lymphoma for whom the first mobilization trial was successful.

**Figure 2:**
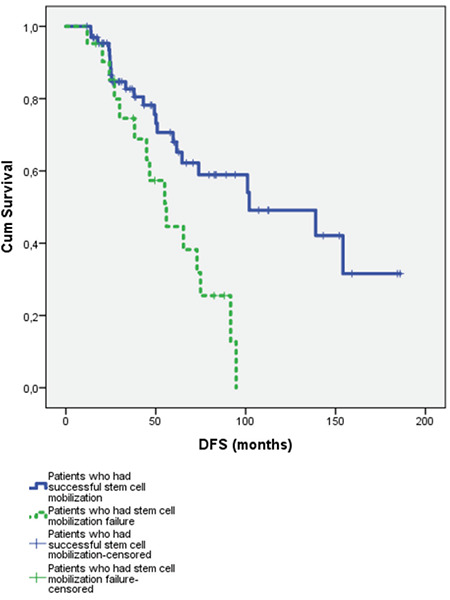
Disease-free survival (DFS) of patients who had successful stem cell mobilization and patients who had stem cell mobilization failure (p=0.004).

The disease-free survival (DFS) rate for patients who had successful stem cell mobilization was 111.9±10.6 months versus 57.6±6.4 months for patients who had stem cell mobilization failure for the first trial; this was a statistically signiﬁcant difference, as shown in Figure 2 (p=0.004). The 3-year DFS rates for patients with successful stem cell mobilization and those with stem cell mobilization failure for the first trial were 82% and 74%, respectively. The 5-year DFS rates for patients with successful stem cell mobilization and stem cell mobilization failure for the first trial were 68% and 44%, respectively.

## Discussion

Stem cell mobilization is still difficult in a significant proportion of patients with lymphoma and the factors predicting poor mobilization are still not fully explained. An obvious reason for these difﬁculties might be the fact that previous studies have been heterogeneous concerning diagnosis, prior therapy, and mobilization regimen used [7]. The frequency of mobilization failure was 24.1% in the ﬁrst mobilization in this study, but no factor was detected in analysis that would cause mobilization failure in these lymphoma patients. No statistically significant difference was found between age, sex, stage of diagnosis, ECOG PS, bone marrow infiltration at diagnosis, induction chemotherapy, chemotherapy cycles before stem cell mobilization, disease status before ASCT, receiving radiotherapy before mobilization, lymphoma types, or mobilization regimen in the two groups. On the other hand, OS and DFS were significantly longer in the group with successful mobilization in the first trial. It was observed that survival outcomes were worse in patients who needed plerixafor for mobilization. However, it was thought that the worse survival outcomes might have been due to the poor bone marrow reserve and disease status before ASCT in patients who needed plerixafor for mobilization.

For successful ASCT, one of the most important factors is to mobilize sufficient numbers of CD34+ cells. In this study, the cut-off value of 2x10^6^ CD34+ cells/kg body weight was determined as the target for a successful mobilization procedure. It can be thought that the necessity of using plerixafor can be predicted according to the number of peripheral CD34 cells. CD34 cell count on apheresis day was reported to be the best predictor of mobilization failure [10]. Additionally, CD34 cell count was suggestive of preemptive plerixafor use and the authors suggested a low level of CD34+ in peripheral blood on day +13 as a possible criterion for initiating plerixafor administration [11]. In this study, the number of CD34+ cells of the apheresis product was observed to be significantly higher in patients who were successful in the first mobilization.

Recent studies reported that the incidence of mobilization failure in lymphoma was as high as 46% [12,13,14]. Variables already reported to be associated with mobilization failure include age, body weight, diagnosis, type of lymphoma and dose of chemotherapy, extent of cell recovery from chemotherapy, bone marrow involvement of lymphoma cells, prior radiation therapy, and interval from diagnosis to mobilization [12,13,14,15]. On the other hand, some hematological parameters such as cytopenia at any stage of mobilization, high mean corpuscular volume, long myelosuppression between salvage chemotherapies, and poor bone marrow microenvironment can predict mobilization failure. Özkurt et al. [16] reported that the CD34+ cell count of the first apheresis product was positively correlated with the white blood cell count, platelet count, peripheral CD34+ cell count, and grade of bone marrow reticulin fibrosis. In this study, chemotherapy-based mobilization was seen to be superior to G-CSF mobilization. Additionally, the platelet count before mobilization was higher in patients who had successful stem cell mobilization than in patients with stem cell mobilization failure. Apart from these two prognostic factors, none of the patient or disease characteristics that we analyzed were associated with mobilization failure. Prognostic factors such as patient characteristics (age, gender, diagnosis, bone marrow involvement, previous number of chemotherapy lines, previous radiotherapy) were also not found to be associated with mobilization failure in previous clinical studies [12,14].

It is not clear whether patients with treatment efficiency may be best mobilized by higher doses of chemotherapy and/or G-CSF. Previously, some studies demonstrated the superiority of chemotherapy plus growth factors over growth factors alone for mobilization [6,17,18]. On the other hand, Pusic et al. [17] found similar rates of mobilization failure with chemotherapy plus growth factors and only growth factor. Additionally, André et al. [19] found no significant difference in CD341 cell harvest yields among 131 patients randomized to receive 5 or 10 µg/kg/day of G-CSF following mobilization chemotherapy. In our study, it was observed that mobilization regime did not affect mobilization failure. However, when the patients who received only G-CSF and those who received a chemotherapy-based mobilization protocol were evaluated, chemotherapy-based mobilization was superior.

## Conclusion

In this study, the success rate of the first mobilization trial was found to be higher in patients with high platelet counts before mobilization and in patients who received chemotherapy-based mobilization protocols. This study had a few limitations. First, it was retrospective. Second, all patients did not receive the same induction chemotherapy before mobilization. Third, the diagnoses of the patients were very heterogeneous. For the patients who had mobilization failure in the first trial, plerixafor was used in a later mobilization, and those patients then had an adequate amount of stem cells for ASCT. Parameters predicting mobilization failure would allow for a preemptive, more cost-effective use of such agents during the ﬁrst mobilization attempt. However, the risk factors for mobilization failure are still not clear.

## Figures and Tables

**Table 1:**
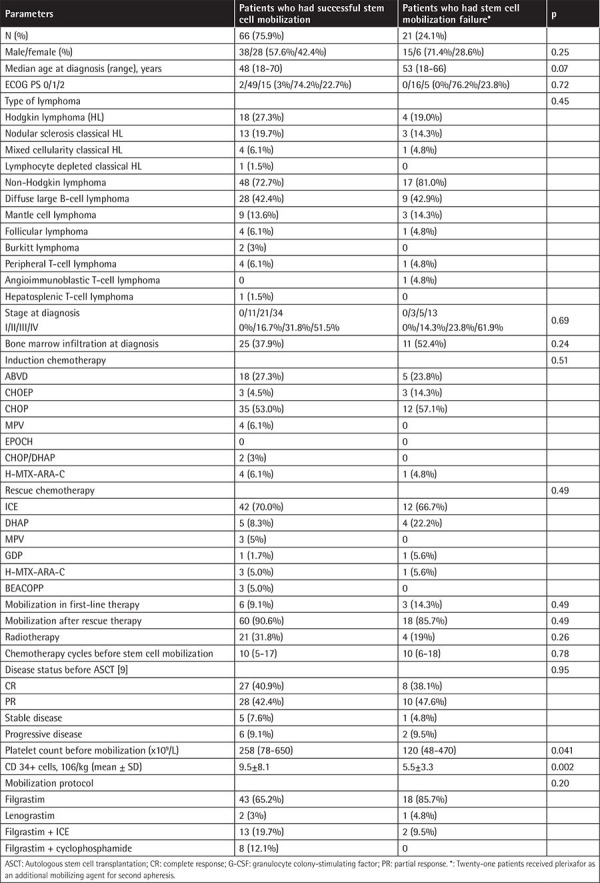
Baseline clinical and demographic characteristics of patients.

**Table 2:**
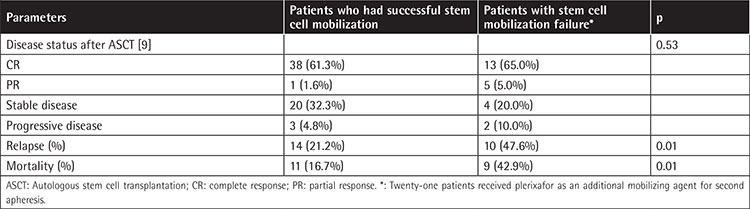
Post-transplantation outcomes.
